# Impact of *Vgsc-*1014 mutations on the feeding pattern of *Phlebotomus argentipes*

**DOI:** 10.1371/journal.pone.0323802

**Published:** 2025-05-28

**Authors:** Bruno Gomes, Debashis Ghosh, Rajib Chowdhury, M. Mamun Huda, Abdul Alim, Mark Paine, Dinesh Mondal, David Weetman

**Affiliations:** 1 Department of Vector Biology, Liverpool School of Tropical Medicine, Liverpool, United Kingdom; 2 Laboratório de Bioquímica e Fisiologia de Insetos, Instituto Oswaldo Cruz (IOC-Fiocruz), Rio de Janeiro, Brazil; 3 International Centre for Diarrhoeal Disease Research (ICDDR), Dhaka, Bangladesh; Tehran University of Medical Sciences, IRAN, ISLAMIC REPUBLIC OF

## Abstract

*Knockdown resistance alleles* (*kdr* alleles) within the para voltage-gated sodium channel gene (*Vgsc*) are a common mechanism of DDT and pyrethroid resistance in insect vectors. In the primary Asian visceral leishmaniasis vector, *Phlebotomus argentipes*, two *kdr* alleles in codon 1014 of the *Vgsc* are associated with insecticide resistance, potentially presenting challenges to vector control efforts in the Indian subcontinent. Here, we screened *Vgsc*-1014 alleles and blood meal origin in *P. argentipes* females collected between September 2013 and August 2015 in Bangladesh (Mymensingh), to understand how *Vgsc*-1014 alleles could impact feeding patterns. The sand fly collection took place in parallel with the vector control agency’s biannual indoor residual spraying (IRS) programme. In this region, the wild-type leucine (*wt*-leucine) was the most common allele (66.7%), followed by the mutant serine (19.4%) and phenylalanine alleles (13.9%). Only 55 sand fly blood meals (13%) came from humans, with most of bovine origin (61%). However, sand flies that had fed on humans showed strongly contrasting *Vgsc*-1014 genotypic frequencies compared to those feeding on other blood sources. Whilst most (81%) *P. argentipes* with human blood possessed *kdr* genotypes with two mutant alleles, most (81%) sand flies feeding on other blood sources possessed genotypes with *wt*-leucine alleles (*P* < 0.001). Significant spatial variation in *kdr* frequencies was detected, but there was no clear temporal trend nor effect of sampling year on any results, and no significant impact of recent IRS in any analyses. The association between human feeding and *kdr* alleles in parallel with pyrethroid spraying indicates a new mechanism of how *kdr* alleles might impact VL control programs.

## Introduction

Visceral leishmaniasis (VL), also known as kala-azar, is a deadly parasitic disease caused by *Leishmania spp*. A large proportion of human cases occur in the north east of the Indian subcontinent, where, via a tripartite agreement between Bangladesh, India, and Nepal, there is a drive toward elimination goals of 1 VL case per 10,000 population in any upazila, block and district respectively in Bangladesh, India and Nepal [1]. In Bangladesh, VL re-appeared in the early 1990s, but ten years of elimination measures reduced VL cases from 9,379 in 2006 to only 168 in 2016 [2]. Bangladesh achieved the elimination target in all upazilas (*i.e.,* administrative division functioning as a sub-unit of a district) in 2017, is sustaining the achievement and obtained WHO certification in 2023 as the first country globally which eliminated VL as a public health problem [3].

The sand fly *Phlebotomus argentipes* is the only known vector of VL in the Indian subcontinent and has no known animal reservoir, which are crucial elements in the feasibility of regional VL elimination [4,5]. Visceral leishmaniasis control programs in other endemic areas present more technical challenges due to their intricate VL transmission cycle with multiple vectors and vertebrate hosts [6,7]. However, *P. argentipes* is considered an endophilic opportunistic biter [8,9], predominately of mammals, with wild-caught female *P. argentipes* most commonly found to have taken bovine or human blood meals [9–12].

Control of *P. argentipes* has been essential to achieve the drastic reductions of VL cases in India [13] and Bangladesh [2,14]. In 2012 indoor resting spraying (IRS) was re-introduced for sand fly control as part of the leishmaniasis control programme in Bangladesh. The programme targeted eight VL-endemic upazilas, five within the Myrmensingh district, for biannual IRS using deltamethrin WP (wettable powder) of household living areas and cattle sheds where present [4,15]. A similar IRS programme in India, has used the pyrethroid alpha-cypermethrin following replacement of DDT in 2016 owing to concerns over resistance [16]. In Bangladesh, IRS has also been complemented by pyrethroid-treated long lasting insecticidal bednets (LLINs) [2]. However, control measures based on pyrethroids (or DDT) may be impacted by insecticide resistance associated with knockdown resistance alleles (*kdr* alleles) within the para voltage-gated sodium channel (*Vgsc*), the target for each insecticide [17]. Substantial resistance to DDT is present in Indian *P. argentipes*, but pyrethroid resistance has yet to be documented [16]. A large-scale study from Nepal detected both DDT and pyrethroid resistance in bioassays [18], albeit using *Anopheles* diagnostic doses, which are lower than those recently established for *P. argentipes* [19], raising concerns for the sustainability of pyrethroid-based control tools.

A study in Bihar (India) screened the *Vgsc* in *P. argentipes* and identified two *kdr* alleles in codon 1014 (L1014F and L1014S). Each is significantly associated with DDT resistance, and with tolerance to pyrethroids [17,20]. Though 1014F confers somewhat stronger DDT-resistance and pyrethroid-tolerance than 1014S, two mutant alleles (FF, FS or SS) appeared to be required for a resistant phenotype in most cases, giving a pragmatic binary separation into *kdr* and non-*kdr* genotypes as resistance markers [17]. Such markers are valuable for molecular surveillance in sand flies, for which, owing to their lab-intractability and sensitivity to rearing conditions, broad-scale phenotypic assessment of insecticide resistance is challenging. Both *kdr* alleles have also been found in *P. argentipes* in West Bengal, and more broadly across north-eastern India [21], Sri Lanka [22,23] and Bangladesh [24], where notably higher frequencies were documented in an area of prolonged IRS implementation (Myrmensingh) than a second geographically-distinct area (Pabna).

In the present study, a temporal sample of *P. argentipes* females from Mymensingh, Bangladesh was screened for *Vgsc-*1014 alleles and blood meal origin to: i) assess *kdr* allele frequencies and their temporal variation in relation to the IRS programme ii) identify the mammalian hosts of *P. argentipes* females and their temporal variation; and iii) determine the association between feeding choice and insecticide resistance, assessed via *kdr* markers.

## Material and methods

### Sample collections

**Fig 1:**
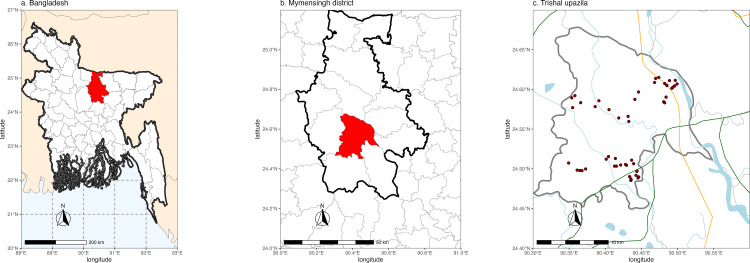
Map with sampling collections in Mymensingh (Bangladesh). Map displayed by R with shapefiles stored in DIVA GIS website (http://www.diva-gis.org/gdata). (A) Bangladesh, (B) Mymensingh district, (C) Trishal upazila.

Indoor residual spraying of sand fly collections took place between September 2013 and August 2015 in 54 sampling sites distributed across 14 localities in the Trishal upazila (sub-district), of Mymensingh district, Bangladesh (Fig 1 and S1 Table). Two sampling methods were used: i) CDC light traps; and ii) indoor resting collections inside human dwellings and cattle sheds using manual aspirators and torches. One CDC light trap was set up, in the corner of the main room for sleeping, close to the wall and 15 cm from the floor, for two consecutive nights between 18:00 and 06:00 hours. All blood-fed *P. argentipes* females were stored individually in absolute ethanol and maintained at -20°C. DNA extraction from individual *P. argentipes* was performed using the ChargeSwitch® Forensic DNA Purification Kit (Thermo Fisher Scientific Inc., MA, USA).

The region has a tropical wet climate (class Aw, Köppen Classification System) [25]. Monthly daily temperatures average between 18.1 °C and 27.6 °C and relative humidity between 61% and 86%. Monthly averages of daily rainfall fluctuates between 9 and 510 mm (https://en.climate-data.org). The monsoon season starts in June and continues until October, and the dry season begins in mid-November and continues to early March [2].

### Genotyping mutations in the voltage-gated sodium channel

The four alleles of *Vgsc*-1014 were genotyped by two TaqMan® SNP genotyping assays [17]. TaqMan reactions were performed in 10 μl volumes containing 1X SensiMix (Bioline, UK), 800 nM each primer, and 200 nM each probe on an Mx3005P qPCR thermal cycler (Agilent Technologies, CA, USA) with initial denaturation of 10 min at 95 °C followed by 40 cycles of 15 s at 92 °C and 1 min at 60 °C.

### Bloodmeal analysis

Identification of bloodmeal origin in *P. argentipes* females was carried out using two PCR assays. The first assay [26] distinguished samples containing mammalian blood from samples with other vertebrate blood. Each amplification was performed individually in a 50 μl PCR reaction that contained 10X DreamTaq Green reaction buffer (Thermo Fisher Scientific), 2.5 mM MgCl_2_, 0.25 mM of each dNTP, 0.25 μM of each primer, and 1U of DreamTaq DNA polymerase (Thermo Fisher Scientific). Thermocycling conditions included an initial denaturation step of 5 min at 95°C followed by 35 cycles each of 95°C for 45 s, 51°C for 60 s, and 72°C for 120 s, and a final extension step of 72°C for 10 min. The second PCR assay [27] was used to categorise blood as originating from four domestic mammals (cow, dog, goat, pig) and humans. Samples positive for mammalian blood in the first assay, but without amplification in the second PCR assay were considered as “other mammalian”. Each amplification was performed individually in a 25 μl PCR reaction that contained 10X DreamTaq Green reaction buffer (Thermo Fisher Scientific), 2 mM MgCl_2_, 0.20 mM of each dNTP, 0.20 μM of each primer, and 1U of DreamTaq DNA polymerase (Thermo Fisher Scientific). Thermocycling conditions included an initial denaturation step of 5 min at 95°C followed by 40 cycles each of 95°C for 60 s, 56°C for 60 s, and 72°C for 60 s, and a final extension step of 72°C for 7 min. All amplifications were carried with positive controls for all five species. Amplification products from both PCR assays were visualized by GelRed™ (Biotium, CA, USA) or peqGREEN (VWR, UK) in 2% agarose gel.

### *Leishmania* infection

*Leishmania* infection in sand flies was screened by a PCR assay [28] with the primers LINR4 (5’ – GGGGTTGGTGTAAAATAGGG – 3’) and LIN19 (5’ – CAGAACGCCCCTACCCG – 3’). Each amplification was performed individually in a 50 μl PCR reaction that contained 10X DreamTaq Green reaction buffer (Thermo Fisher Scientific), 2 mM MgCl_2_, 0.40 mM of each dNTP, 0.20 μM of each primer, and 1U of DreamTaq DNA polymerase (Thermo Fisher Scientific). Thermocycling conditions included an initial denaturation step of 5 min at 94°C followed by 35 cycles each of 94°C for 30 s, 62°C for 30 s, and 72°C for 60 s, and a final extension step of 72°C for 10 min. Samples with positive amplifications were confirmed by an ITS-1 PCR assay specific for *Leishmania* [29]. Each amplification was performed individually in a 50 μl PCR reaction that contained 10X DreamTaq Green reaction buffer (Thermo Fisher Scientific), 2 mM MgCl_2_, 0.40 mM of each dNTP, 0.40 μM of each primer, and 1U of DreamTaq DNA polymerase (Thermo Fisher Scientific). Thermocycling conditions included an initial denaturation step of 5 min at 94°C followed by 35 cycles each of 94°C for 30 s, 53°C for 30 s, and 72°C for 60 s, and a final extension step of 72°C for 10 min. All amplifications were carried with two *Leishmania* positive controls. Amplification products from both PCR assays were visualized by GelRed™ (Biotium, CA, USA) or peqGREEN (VWR, UK) in 2% agarose gel.

### Data analysis

A chi-square test was used to assess associations between *Vgsc*-1014 allele/genotype frequencies and other traits collected: 1) sampling data (*i.e.,* region, locality, endemicity); and 2) the vertebrate origin of blood meals. We used the R Scripts associated with the software POPS [30] to display spatial interpolations of *Vgsc*-1014 allele frequencies applying kriging techniques, while collection sites were mapped using the R-packages “sf”, “ggplot2”, “rnaturalearth”, “rnaturalearthdata”, “ggspatial” with shapefiles stored in DIVA-GIS website (http://www.diva-gis.org/gdata). We ran Generalized Linear Models with a binary logistic link function in Stata 16 to test the effect of potentially influencing factors on two types of dependent variables. 1) the effect of region (north vs south) to reflect a natural geographical split in the villages sampled, leishmaniasis endemicity (classified as high, moderate and low by the vector control programme), collection year (pragmatically divided into years 1 or 2 since sample size precluded more granular temporal analysis), IRS spraying within the past 3 months (yes or no), collection method (CDC light trap or manual aspiration), collection in cattleshed (yes or no) on the presence of a *kdr Vgsc*-1014 genotype (*kdr* or not *kdr*). 2) the effect of the same independent variables but with the omission of year of sampling (because of an extreme asymmetry across years) and addition of the presence of a *kdr Vgsc*-1014 genotype (*kdr* or not *kdr*) on (a) human blood feeding and (b) cow blood feeding. In each model village was included as a random variable. Except where noted above (for GLM 2) all available variables of potential interest were included as factors in the models, without prior selection. These data were collected as a routine part of the monitoring programme and were not specifically selected for the current analyses.

## Results

A total of 648 *P. argentipes* blood-fed females from Mymensingh region (Bangladesh) were genotyped for the *Vgsc*-1014 locus. The wild-type leucine allele was the most common (66.7%), but all three mutant alleles were present, with a slightly higher frequency of serine (TCA, 19.4%) than phenylalanine alleles (TTT or TTC, 13.9% combined frequency).

Allelic frequencies differed significantly between northern and southern villages in the upazila (Table 1; *χ*^2^_2_ = 280.1; *P *< 0.001). Southern villages exhibited lower levels of the wild-type leucine than mutant alleles (Leu frequency < 50%), and villages with different visceral leishmaniasis (VL) endemicity did not show significant differences in *Vgsc*-1014 alleles (*χ*^*2*^_*4*_ = 1.346; *P *= 0.854). In contrast, northern villages showed a mixed scenario where most villages exhibited lower *kdr* levels than the south, and significant differences were observed among villages with different endemicity (*χ*^*2*^_*4*_ = 35.42; *P *= 3.8 × 10^-7^; Table 1), although this could be an artifact of asymmetric sampling, caused by the very large sample from Khustia-2.

**Table 1:** Allelic frequency of *Vgsc*-1014 by geographic information.

Region	Village	VL Endemicity	*N*	S		F		L	
				*N*	*f*	*N*	*f*	*N*	*f*
NORTH	KANIHARI	High	32	13	(40.6)	6	(18.8)	13	(40.6)
	KHUSTIA-2	High	586	27	(4.6)	27	(4.6)	532	(90.8)
	KAKCHOR	Moderate	6	2	(33.3)	0	(0.0)	4	(66.7)
	KHUSTIA-1	Moderate	26	3	(11.5)	0	(0.0)	23	(88.5)
	SOLIMPUR	Moderate	16	2	(12.5)	1	(6.2)	13	(81.2)
	BIEARTA	Non/Low	24	1	(4.2)	4	(16.7)	19	(79.1)
	BIRRAMPUR	Non/Low	14	4	(28.6)	4	(28.6)	6	(42.8)
	CHORPARA	Non/Low	4	2	(50.0)	2	(50.0)	0	(0.0)
	TOTAL	–	708	54	(7.6)	44	(6.2)	610	(86.2)
SOUTH	CHAOLADI	High	110	46	(41.8)	27	(24.6)	37	(33.6)
	GOLAVITA	High	170	51	(30.0)	39	(22.9)	80	(47.1)
	RAEARGRAM	Moderate	58	18	(31.0)	13	(22.4)	27	(46.6)
	KANDAPARA	Non/Low	140	47	(33.6)	31	(22.1)	62	(44.3)
	KHAGATI	Non/Low	60	26	(43.3)	19	(31.7)	15	(25.0)
	TOTAL	–	538	188	(34.9)	129	(23.9)	221	(41.1)

*N*: number of alleles; *f*: relative frequencies (in percentage) of each allele; S: number serine allele (TCA); F: number of phenylalanine alleles (TTC and TTT); L: number of leucine allele (TTA, wild type).

**Fig 2:**
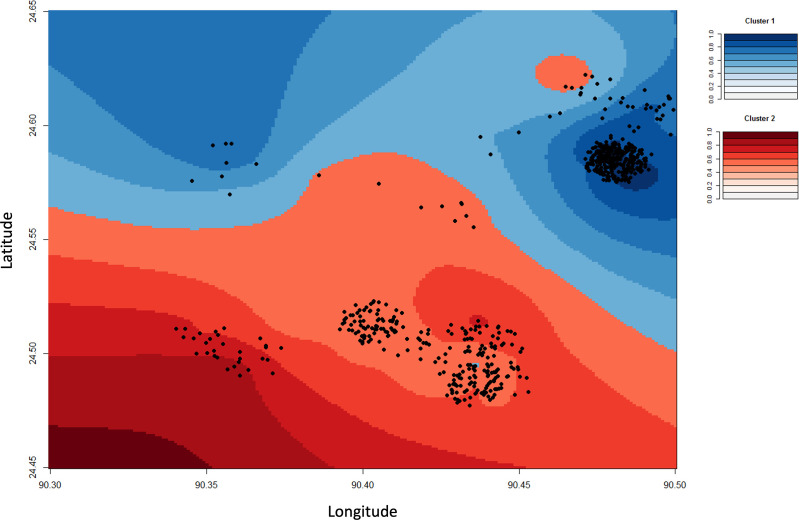
Spatial projection of *vgsc-*1014 alleles. Blue: Leucine alleles (wild type); Red: *Knockdown* mutations associated with resistance (serine and phenylalanine).

Spatial projections illustrate the higher *wt-*leucine frequencies in northern villages, whilst highlighting the over-representation of samples from the northeast (*i.e.,* Khustia-2). *Kdr* alleles are consistently more frequent in southern villages (Fig 2). There was no clear variation in *Vgsc* genotype frequencies across time, with no directional temporal trend (S1 Fig).

Subsequent analyses grouped genotypes as those predicted to be phenotypically susceptible (any genotype containing a wild type allele, leucine) or resistant (genotypes with two *kdr* alleles, whether serine or phenylalanine) [17]. A GLM, which accounts for village as a random variable, was applied to investigate the effects of several variables on *kdr*. This confirmed the significant difference between northern and southern regions but all other factors (endemicity, year, IRS, collection method, and collection from cattleshed) were non-significant (S2 Table). This suggests that the significant result above for variation in *Vgsc*-1014 alleles with endemicity in northern villages resulted from sampling asymmetries, which were controlled by addition of village as a random factor.

**Fig 3:**
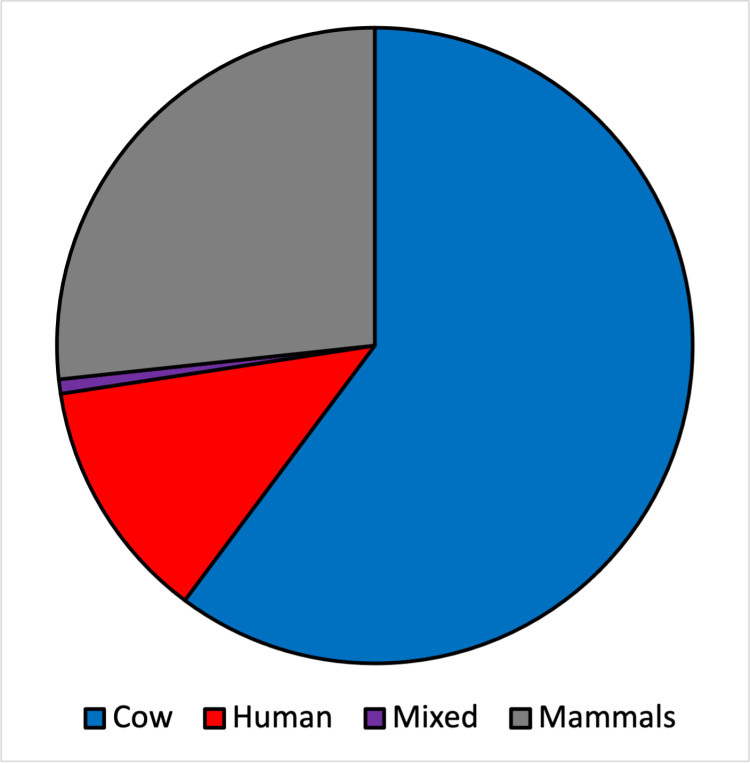
Animal hosts from *Phlebotomus argentipes* blood meals by species ID assay (N = 410). Blue: cow; Red: human; Purple: mix feeds with human and cow; Grey: other mammals that include samples without positive band in species ID and samples with pig and goat blood.

A total of 410 blood meals were positive for mammalian blood (63% of the samples). Most of these blood meals contained cow blood (61%; N = 252), while 51 sand flies (12%) had fed only in humans. Four sand flies had mixed meals from both human and cow blood, three from alternative domestic animals (2 in goat, 1 in pig), and the remaining samples were positive for mammalian blood but did not yield a species-band in the assay (Fig 3).

Sand flies with human blood meals (*N *= 55) showed strongly contrasting *Vgsc*-1014 genotypic frequencies compared to those without human blood (Table 2). The *P. argentipes* with human blood exhibited primarily *kdr* genotypes (81%), which also includes a substantial fraction of genotypes with phenylalanine (52%; Table 2), which is known to associate more strongly with resistance phenotype. In contrast, sand flies without human blood showed a high frequency of genotypes with the *wt-*leucine allele (81%; Table 2) that are considered susceptible [17].

**Table 2:** Association of blood meal source (cow or human) with knockdown resistance (*kdr*) mutations in *P. argentipes.*

	Leu/*	Ser/Ser	Ser/Phe	Phe/Phe	Odds Ratio	*P*-value
Non-human	290	18	35	12	29.01	<0.001
Human	10	17	21	7		

*Kdr* genotypes are: Ser/Ser, Ser/Phe, Phe/Phe; all other genotypes (containing a wild type Leu allele) are classed as susceptible. Odds ratios and *P*-values from a GLM modelling predictors of human blood feeding are shown; full model results are shown in S3 Table.

A GLM of human feeding showed a strong impact of *kdr* genotype, i.e., a positive association between human feeding and presence of *kdr* (S3A Table). A GLM of cow feeding also showed a highly significant effect of *kdr*, but with a negative association, i.e., flies with cow feeds had more non-*kdr* genotypes (S3B Table). Other factors in both models (region, endemicity, IRS, cattleshed collection) were not significant, with the exception of collection method in the human-bloodfeeding GLM, which showed significantly higher *kdr* in flies collected by manual aspiration than those collected in CDC light traps (S3A Table).

**Fig 4:**
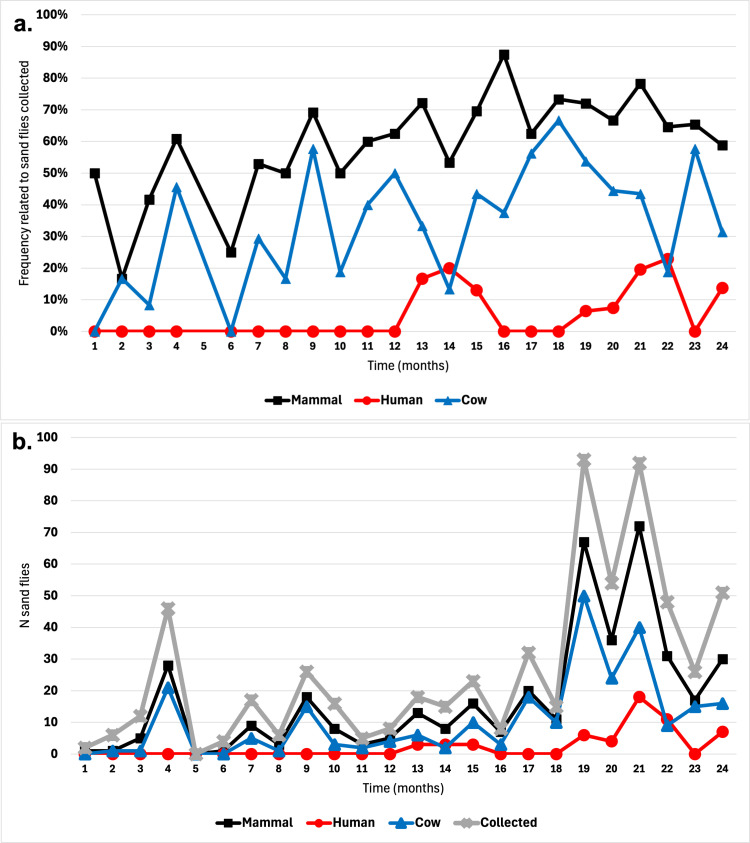
Variation of blood meal origin across 24 months of collections. (A) Frequency related with sand flies collected across time. (B) Number of sand flies collected across time. Note that no sand flies were collected in month 5.

A striking observation is the lack of human blood meals recorded before the twelfth month of the study while human feeding frequency presents a bimodal distribution in the second year, which was observed in both regions (human *vs.* cow between years: North, *χ*^*2*^_*1*_ = 4.64; *P *= 0.031; South, *χ*^*2*^_*1*_ = 6.04; *P *= 0.014; Fig 4).

All the 648 *P. argentipes* were negative for *Leishmania* infection when screened by molecular assays. All the assays were carried out with positive controls from laboratory *Leishmania spp.* cultures.

## Discussion

In this study, a notable difference in *kdr*-1014 genotypes was found between sand flies that fed on humans and on non-human mammal hosts. The higher frequencies of *kdr* genotypes (81%) in the 55 *P. argentipes* females with human blood (13% of total samples with mammal blood) contrast with the incidence of *wt-*leucine alleles (> 80%) in most sand flies that only fed on bovine (61% of samples with mammal blood) or other mammals (26% of samples with mammal blood). Whilst the pronounced impacts of *kdr* mutations in altering the excitability of the nervous system may have widespread effects, impacts on bloodfeeding behaviour are scarce in the literature [31]. Host seeking behaviour in pyrethroid-resistant *Anopheles gambiae* s.s. were slightly different than susceptible populations [32].

Effects of *Vgsc* mutations on behaviour have been observed in aphids and houseflies, where lower responses to olfactory cues and responses to thermal gradients, respectively [31]. Moreover, *Vgsc* variation in sand flies have been also associated with different courtship songs involved in the speciation process of two lineages of *Lutzomyia longipalpis* complex [33,34], however, none of the variants involved in this study are known *kdr* mutations. An alternative explanation for the low proportion of human feeding in sand flies with susceptible genotypes could be more efficient killing of these sand flies by control methods targeted at human dwellings – which could come from multiple sources, rather than just IRS - or inducing them to avoiding human biting, which could involve the emergence of behavioural resilience [35]. This latter mechanism could represent a behaviour shift within the pre-establish biting behaviour of vectors that promote the evasion of indoor control methods such as observed in malaria vectors of the *Anopheles gambiae* complex [35]. The increase of outdoor biting and shift in the biting time of this species complex brings concerns about the sustainability of the malaria control programs associated with LLINs/IRS in Africa [36,37]. Moreover, if outdoor biting increases for *P. argentipes,* this will probably increase the odds of biting cattle due to cattle’s higher exposure during their feeding period than humans. In the literature, a drastic overall reduction of human feeding from 30% to 9% occurred in Bihar after DDT spraying that includes different housing facilities: i) human houses: from 66% to 32%; ii) animal shelters: from 11% to 2%; and iii) mixed dwellings: 30% to 8% [9].

Overall, we found a higher proportion of bovine blood meals (61%) in Mymensingh (Bangladesh), which is common in populations of *P. argentipes* from the Indian subcontinent [9,11]. This feeding tendency is consistent with baited/landing collections where a higher proportion of *P. argentipes* was caught in cattle than humans [38]. In fact, older records usually describe *P. argentipes* as primarily zoophilic with some human biting [39]. Only later in the 1970s and 80s, was more consistently anthropophilic behaviour observed in *P. argentipes* of West Bengal (India) primarily associated with human dwellings [40,41]. Recent studies in Nepal and northern districts of Bihar (Saran and Muzaffarpur) indicate a higher proportion of wild-caught sand flies with human blood (69% - 81%) than other mammals and a high proportion of sand flies with multiple host blood (30% - 60%) in Bihar [8,10,12]. A high variation in bovine/human feeding ratio is not only observed across broad geographic regions because feeding patterns of *P. argentipes* can also vary among local collection sites [9,42].

Perhaps unsurprisingly, a higher proportion of human feeding is usually found in human dwellings (65% - 69%), while cattle shed collections yield a higher proportion of bovine feeding (78% - 92%). Nevertheless, a study in Bihar [12] shows a higher frequency of human blood meals than cattle in all habitats along with a high proportion of multiple hosts per sand fly. These studies also consistently indicate a higher collection of sand flies in cattle sheds than human houses suggesting the higher attractivity of *P. argentipes* for cattle than humans [9,12,42]. In this study, we did not find any influence of whether samples were collected from cattlesheds or human dwellings on whether they had taken a human blood meal, but we could infer the impact of other factors. Although outdoor collections were not included in the main analyses because of small sample size, of the 29 blood-fed sand flies collected outside houses, none had fed on humans, whereas 66% of blood meals were from cattle. Moreover, we did not find a significant difference in host blood origin (*χ*^*2*^_*2*_ = 2.125; *P *= 0.346) or *Vgsc*-1014 alleles (S2 Table) between the two collection methods (manual collections and CDC traps). Further studies will be necessary to clarify the variation of bovine/human feeding ratio for *P. argentipes* associated with ecological characteristics (e.g., dwelling types, environmental factors) and genetic characteristics (i.e., *kdr* alleles).

All polymorphisms of *Vgsc*-1014 described in Bihar (India) are present in Mymensingh but with different frequencies [17]. The *wt*-leucine allele was the most frequent in both regions of Mymensingh (41.1% - 86.2%), contrasting with the lower frequency found in Patna and Vaishali in Bihar (54.% - 23.3%) [17], West Bengal (22.3%) [22]. However, the combination of both *kdr alleles* was higher than the susceptible allele’s frequency in southern Mymensingh (Fig 2), similar to the scenario described in Sri Lanka [23]. Phenylalanine alleles (i.e., the stronger resistant/tolerant phenotype) show an inverse scenario compared to *wt-*leucine alleles, with the lowest frequency in Mymensingh (6.2% - 23.9%), but frequencies of 53.9% and 50.9% in Vaishali (Bihar) and Sri Lanka, respectively. Serine alleles show higher frequency in Patna/Vaishali (40.7% - 47.7%), West Bengal (50.5%), and the southern localities of Mymensingh (34.9%) [17,22,23]. The lower proportion of *kdr* alleles in Mymensingh, particularly in northern localities, indicates a lower potential for DDT and pyrethroid resistance in this region when compared with Bihar. This scenario is consistent with the early success in Bangladesh, which achieved the VL elimination target in all upazilas [43], and challenges associated with DDT resistance in India that required an insecticide change in the spraying control strategy [44]. The lack of temporal trends of *Vgsc*-1014 alleles in Mymensingh indicate a stable scenario with no increase in *kdr* under active control programs based on pyrethroids, with similar findings from across the VL-endemic region of northern India [21]. However, our studies principal finding of higher human feeding in sand flies with *kdr*-1014 genotypes could suggest an impact on control programmes.

Our study had several limitations. (1) The lack of detection of *Leishmania* parasites in the sand flies collected precluded analysis of any association between *kdr* frequencies and infection. This may have been a result of the moderate sample size (N = 648) but with an upper 95% confidence limit of 0.6% for the prevalence estimate, suggests a very low infection rate in the population, likely reflecting success of the control programme [3]. (2) We cannot rule out additional, unrecorded variables as potential confounding factors affecting the results, e.g., variation in levels of vector control activities applied to different areas. (3) Our analysis found a lack of association between IRS activities in the months before the collections were made, but we exclude the potential effects of previous control activities. (4) The sample size for the feeding preference analysis was moderate with less than half of the samples analysed providing an identifiable blood source. Whilst this did not prevent detection of significant associations between *kdr* frequency and blood feeding it precluded detailed analysis of spatio-temporal trends. However, the significant difference in human vs. bovine blood meals between sampling years, each of which covered the different seasons and present in both northern and southern areas indicates that timing of sampling had effects, which should be considered in future studies. (5) The spatial distribution of the samples collected was very uneven with a far larger collection in one village than others. Once this variation was accounted for in our analysis, we detected only a north vs. south area difference in *kdr* frequencies with all other factors (endemicity, year, IRS, collection method, and collection from cattleshed) non-significant. With a more even spatial distribution of samples, the GLM would have had higher power to detect potential effects of these additional factors. Moreover, it should be noted that the variables included in the model were those collected by the monitoring programme and other important unrecorded factors may have been omitted. (6) The only resistance mechanism we examined was *kdr*, and though evidence for metabolic resistance in *P. argentipes* is very limited [45], we cannot rule out the presence of other resistance mechanisms and possible influences these may have on bloodfeeding.

## Conclusion

The association between human feeding and *kdr* in a region with pyrethroid spraying is of potential medical significance, adding importance for screening *kdr* markers in surveillance and control programs of *P. argentipes*. Pyrethroids are currently the primary insecticide in VL control programs, and the association between feeding and *kdr* genotypes provides further evidence indicating the importance of finding alternative control measures.

## Supporting information

S1 Table Geographic information from *P. argentipes* blood fed females. *N*: total number of *P. argentipes* females per sampling point (10 sand flies from K2U01 were collected outside the houses, while all other sand flies were collected inside houses); Spray: date when control programme sprayed deltamethrin.
(DOCX)

S2 Table GLM analysis of *kdr* genotype (*kdr*/no *kdr*). This analysis was carried based on resistance phenotype of the sand flies: 1) phenotypically susceptible (any genotype containing a wild type allele, leucine) vs. 2) phenotypically resistant (genotypes with two *kdr* alleles, whether serine or phenylalanine).
(DOCX)

S3 Table GLM analysis of blood meal sources. (A) Human-biting. (B) Cow-biting. *Kdr*: “no” corresponds to genotypes with at list one Leucine alelle, and “yes” corresponds to genotypes with two *kdr* alleles.” Year is omitted from the models because of lack of human biting in year 1 of the study.
(DOCX)

S1 Fig Variation of the *kdr* genotypes across time. Note that no sand flies were collected in month 5.
(DOCX)
