# Supplementary material for: Impact of Vgsc-1014 mutations on the feeding pattern of Phlebotomus argentipes
Source: PLoS One. 2025 May 28;20(5):e0323802. doi: 10.1371/journal.pone.0323802 (PMC12118823; doi:10.1371/journal.pone.0323802)
Supplement: S1 Table — N: total number of P. argentipes females per sampling point (10 sand flies from K2U01 were collected outside the houses, while all other sand flies were collected inside houses); Spray: date when control programme sprayed deltamethrin. (DOCX) [file pone.0323802.s001.docx]

**S1 Table. Geographic information from *P. argentipes* blood fed females.**

| **Region** | **Village** | **Endemicity** | **Sampling point** | **Latitude** | **Longitude** | ***N*** | **Spray BT** | **Spray 1** | **Spray 2** | **Spray 3** |
| --- | --- | --- | --- | --- | --- | --- | --- | --- | --- | --- |
| NORTH | KANIHARI | High | KAI01 | 24.607389 | 90.482139 | 2 | 04/05/13 | 25/12/13 | 24/06/14 |  |
|  |  |  | KAI02 | 24.608667 | 90.468167 | 3 | 04/05/13 | 24/06/14 | 24/03/15 |  |
|  |  |  | KAI03 | 24.613750 | 90.469611 | 1 | 04/05/13 | 25/12/13 | 24/06/14 |  |
|  |  |  | KAI04 | 24.615028 | 90.473972 | 10 | NA | 24/06/14 | 24/03/15 |  |
|  | KHUSTIA-2 | High | K2U01 | 24.584306 | 90.480861 | 221 | 09/05/13 | 25/12/13 | 18/07/14 |  |
|  |  |  | K2U02 | 24.584361 | 90.480667 | 15 | 09/05/13 | 25/12/13 | 18/07/14 |  |
|  |  |  | K2U03 | 24.590222 | 90.484056 | 11 | 09/05/13 | 25/12/13 | 18/07/14 |  |
|  |  |  | K2U04 | 24.590306 | 90.484028 | 5 | 09/05/13 | 25/12/13 | 18/07/14 |  |
|  |  |  | K2U05 | 24.583139 | 90.482167 | 41 | 09/05/13 | 25/12/13 | 18/07/14 |  |
|  | KAKCHOR | Moderate | KAK01 | 24.587389 | 90.440917 | 1 | 02/05/13 | 25/12/13 | 06/07/14 | 11/03/15 |
|  |  |  | KAK02 | 24.596694 | 90.445944 | 2 | 02/05/13 | 25/12/13 | 06/07/14 | 7/03/15 |
|  | KHUSTIA-1 | Moderate | K1U01 | 24.601361 | 90.492083 | 3 | 09/05/13 | 25/12/13 | 16/06/14 |  |
|  |  |  | K1U02 | 24.602806 | 90.493611 | 2 | 09/05/13 | 25/12/13 | 17/06/14 |  |
|  |  |  | K1U03 | 24.604806 | 90.485222 | 2 | 09/05/13 | 25/12/13 | 16/06/14 |  |
|  |  |  | K1U04 | 24.605250 | 90.485028 | 2 | 09/05/13 | 25/12/13 | 16/06/14 |  |
|  |  |  | K1U05 | 24.611028 | 90.488750 | 4 | 09/05/13 | 25/12/13 | 17/06/14 |  |
|  | SOLIMPUR | Moderate | SOL01 | 24.589417 | 90.353694 | 3 | 12/05/13 | 25/12/13 | 27/06/14 | 11/03/15 |
|  |  |  | SOL02 | 24.592111 | 90.358028 | 1 | 12/05/13 | 25/12/13 | 27/06/14 |  |
|  |  |  | SOL03 | 24.577028 | 90.354917 | 3 | 12/05/13 | 25/12/13 | 27/06/14 |  |
|  |  |  | SOL04 | 24.583278 | 90.365972 | 1 | 12/05/13 |  |  |  |
|  | BIEARTA | Non/Low | BIE01 | 24.604417 | 90.495944 | 1 | 26/04/13 | 25/12/13 | 10/06/14 |  |
|  |  |  | BIE02 | 24.606833 | 90.499333 | 1 | NA | 10/06/14 |  |  |
|  |  |  | BIE03 | 24.611333 | 90.496806 | 10 | NA | 10/06/14 |  |  |
|  | BIRRAMPUR | Non/Low | BIR01 | 24.559667 | 90.432611 | 5 | 26/04/13 | 25/12/13 | 5/07/14 | 29/03/15 |
|  |  |  | BIR02 | 24.564000 | 90.418611 | 1 | 26/04/13 |  |  |  |
|  |  |  | BIR03 | 24.566056 | 90.431556 | 1 | 26/04/13 | 25/12/13 | 5/07/14 |  |
|  | CHORPARA | Non/Low | CHO01 | 24.574500 | 90.405194 | 1 | 26/04/13 | 15/02/14 | 24/03/15 |  |
|  |  |  | CHO02 | 24.578278 | 90.386056 | 1 | 26/04/13 |  |  |  |
| SOUTH | CHAOLADI | High | CHA01 | 24.504417 | 90.429361 | 3 | 28/04/13 | 25/12/13 | 06/07/14 | 12/03/15 |
|  |  |  | CHA02 | 24.504806 | 90.421222 | 4 | 28/04/13 | 25/12/13 | 06/07/14 | 9/03/15 |
|  |  |  | CHA03 | 24.505500 | 90.427917 | 9 | 28/04/13 | 25/12/13 | 06/07/14 | 9/03/15 |
|  |  |  | CHA04 | 24.506472 | 90.439250 | 36 | 28/04/13 | 03/07/14 | 15/03/15 |  |
|  |  |  | CHA05 | 24.510917 | 90.436472 | 3 | 28/04/13 | 25/12/13 | 03/07/14 | 15/03/15 |
|  | GOLAVITA | High | GOL01 | 24.503361 | 90.412806 | 5 | 30/04/13 | 25/12/13 | 14/07/14 | 31/03/15 |
|  |  |  | GOL02 | 24.503361 | 90.415417 | 3 | 30/04/13 | 14/07/14 | 31/03/15 |  |
|  |  |  | GOL03 | 24.512139 | 90.401333 | 53 | 30/04/13 | 25/12/13 | 13/07/14 | 31/03/15 |
|  |  |  | GOL04 | 24.513056 | 90.414000 | 6 | 30/04/13 | 25/12/13 | 14/07/14 | 30/03/15 |
|  |  |  | GOL05 | 24.515389 | 90.404444 | 18 | 30/04/13 | 25/12/13 | 13/07/14 | 30/03/15 |
|  | RAEARGRAM | Moderate | RAE01 | 24.488528 | 90.445333 | 15 | 12/05/13 | 25/12/13 | 27/06/14 | 7/03/15 |
|  |  |  | RAE02 | 24.489806 | 90.446167 | 1 | 12/05/13 | 25/12/13 |  |  |
|  |  |  | RAE03 | 24.491778 | 90.442444 | 1 | 12/05/13 | 25/12/13 |  |  |
|  |  |  | RAE04 | 24.496750 | 90.443750 | 9 | 12/05/13 | 25/12/13 | 26/06/14 | 10/03/15 |
|  |  |  | RAE05 | 24.497278 | 90.443889 | 3 | 12/05/13 | 25/12/13 | 26/06/14 | 10/03/15 |
|  | KANDAPARA | Non/Low | KAN01 | 24.485389 | 90.435444 | 12 | 02/05/13 | 25/12/13 | 24/06/14 | 9/03/15 |
|  |  |  | KAN02 | 24.485417 | 90.435472 | 18 | 02/05/13 | 25/12/13 | 24/06/14 | 9/03/15 |
|  |  |  | KAN03 | 24.486556 | 90.435583 | 22 | NA | 25/12/13 | 24/06/14 | 9/03/15 |
|  |  |  | KAN04 | 24.487417 | 90.435389 | 7 | 02/05/13 | 25/12/13 | 24/06/14 | 9/03/15 |
|  |  |  | KAN05 | 24.490194 | 90.433639 | 11 | 02/05/13 | 25/12/13 | 24/06/14 | 9/03/15 |
|  | KHAGATI | Non/Low | KHA01 | 24.507278 | 90.349333 | 14 | 04/05/13 | 28/06/14 | 28/03/15 |  |
|  |  |  | KHA02 | 24.497778 | 90.367861 | 1 | 04/05/13 | 25/12/13 | 28/06/14 | 28/03/15 |
|  |  |  | KHA03 | 24.497889 | 90.364861 | 3 | 04/05/13 |  |  |  |
|  |  |  | KHA04 | 24.498333 | 90.361167 | 8 | 04/05/13 | 25/12/13 | 28/06/14 | 25/03/15 |
|  |  |  | KHA05 | 24.499806 | 90.372861 | 4 | NA | 25/12/13 | 24/06/14 | 25/03/15 |

*N*: total number of *P. argentipes* females per sampling point (10 sand flies from K2U01 were collected outside the houses, while all other sand flies were collected inside houses); Spray: date when control programme sprayed deltamethrin.
