# Supplementary material for: Impact of Vgsc-1014 mutations on the feeding pattern of Phlebotomus argentipes
Source: PLoS One. 2025 May 28;20(5):e0323802. doi: 10.1371/journal.pone.0323802 (PMC12118823; doi:10.1371/journal.pone.0323802)
Supplement: S2 Table — This analysis was carried based on resistance phenotype of the sand flies: 1) phenotypically susceptible (any genotype containing a wild type allele, leucine) vs. 2) phenotypically resistant (genotypes with two kdr alleles, whether serine or phenylalanine). (DOCX) [file pone.0323802.s002.docx]

S2 Table. GLM analysis of *kdr* genotype (*kdr*/no *kdr*)

|  |  | Odds ratio | 95% LCL | 95% UCL | z | P-value |
| --- | --- | --- | --- | --- | --- | --- |
| **Region** | **north** | **reference** |  |  |  |  |
|  | **south** | **4.911** | **1.166** | **20.685** | **2.17** | **0.030** |
| Endemicity | low | reference |  |  |  |  |
|  | moderate | 0.281 | 0.076 | 1.037 | -1.91 | 0.057 |
|  | high | 0.575 | 0.163 | 2.028 | -0.86 | 0.389 |
| Year | 1 | reference |  |  |  |  |
|  | 2 | 1.361 | 0.610 | 3.038 | 0.75 | 0.452 |
| IRS_past_3_months | no | reference |  |  |  |  |
|  | yes | 1.268 | 0.683 | 2.356 | 0.75 | 0.452 |
| Collection method | CDC-LT | reference |  |  |  |  |
|  | aspiration | 2.114 | 0.250 | 17.854 | 0.69 | 0.492 |
| Cattleshed | no | reference |  |  |  |  |
|  | yes | 1.225 | 0.568 | 2.642 | 0.52 | 0.604 |
| Intercept |  | 0.227 | 0.055 | 0.935 | -2.05 | 0.040 |
|  |  |  |  |  |  |  |
| Village (random) |  | 0.658 | 0.273 | 1.584 |  |  |
|  |  |  |  |  |  |  |

This analysis was carried based on resistance phenotype of the sand flies: 1) phenotypically susceptible (any genotype containing a wild type allele, leucine) vs. 2) phenotypically resistant (genotypes with two *kdr* alleles, whether serine or phenylalanine).
