# Supplementary material for: Impact of Vgsc-1014 mutations on the feeding pattern of Phlebotomus argentipes
Source: PLoS One. 2025 May 28;20(5):e0323802. doi: 10.1371/journal.pone.0323802 (PMC12118823; doi:10.1371/journal.pone.0323802)
Supplement: S3 Table — (A) Human-biting. (B) Cow-biting. Kdr: “no” corresponds to genotypes with at list one Leucine alelle, and “yes” corresponds to genotypes with two kdr alleles.” Year is omitted from the models because of lack of human biting in year 1 of the study. (DOCX) [file pone.0323802.s003.docx]

S3 Table. GLM analysis of blood meal sources

| **A. Human-biting** |  |  |  |  |  |  |
| --- | --- | --- | --- | --- | --- | --- |
|  |  | Odds ratio | 95% LCL | 95% UCL | z | P-value |
| Region | north | reference |  |  |  |  |
|  | south | 0.318 | 0.043 | 2.331 | -1.13 | 0.259 |
| Endemicity | low | reference |  |  |  |  |
|  | moderate | 3.190 | 0.127 | 80.263 | 0.71 | 0.481 |
|  | high | 6.192 | 0.479 | 80.072 | 1.40 | 0.163 |
| IRS_past_3_months | no | reference |  |  |  |  |
|  | yes | 1.635 | 0.633 | 4.224 | 1.02 | 0.310 |
| **Collection method** | **CDC-LT** | **reference** |  |  |  |  |
|  | **aspiration** | **4.666** | **1.169** | **18.618** | **2.18** | **0.029** |
| Cattleshed | no | reference |  |  |  |  |
|  | yes | 2.414 | 0.804 | 7.250 | 1.57 | 0.116 |
| **kdr** | **no** | **reference** |  |  |  |  |
|  | **yes** | **29.085** | **5.914** | **143.036** | **4.15** | **<0.001** |
| Intercept |  | 0.002 | 0.000 | 0.035 | -4.28 | <0.001 |
|  |  |  |  |  |  |  |
| Village (random) |  | 2.006 | 0.713 | 5.640 |  |  |
|  |  |  |  |  |  |  |
|  |  |  |  |  |  |  |
| **B. Cow-biting** |  |  |  |  |  |  |
|  |  | Odds ratio | 95% LCL | 95% UCL | z | P-value |
| Region | north | reference |  |  |  |  |
|  | south | 1.463 | 0.862 | 2.482 | 1.41 | 0.159 |
| Endemicity | low | reference |  |  |  |  |
|  | moderate | 1.192 | 0.452 | 3.140 | 0.36 | 0.722 |
|  | high | 1.344 | 0.700 | 2.579 | 0.89 | 0.374 |
| IRS_past_3_months | no | reference |  |  |  |  |
|  | yes | 1.156 | 0.620 | 2.157 | 0.46 | 0.648 |
| Collection method | CDC-LT | reference |  |  |  |  |
|  | aspiration | 0.787 | 0.423 | 1.464 | -0.76 | 0.450 |
| Cattleshed | no | reference |  |  |  |  |
|  | yes | 0.954 | 0.496 | 1.835 | -0.14 | 0.888 |
| **kdr** | **no** | **reference** |  |  |  |  |
|  | **yes** | **0.094** | **0.054** | **0.165** | **-8.26** | **<0.001** |
| Intercept |  | 0.872 | 0.446 | 1.706 | -0.4 | 0.689 |
|  |  |  |  |  |  |  |
| Village (random) |  | 5.10e-34 | 1.91e-38 | 1.36e-29 |  |  |
|  |  |  |  |  |  |  |

*Kdr*: “no” corresponds to genotypes with at list one Leucine alelle, and “yes” corresponds to genotypes with two kdr alleles.” Year is omitted from the models because of lack of human biting in year 1 of the study.
