# Supplementary figures and images for: Impact of Vgsc-1014 mutations on the feeding pattern of Phlebotomus argentipes
Source: PLoS One. 2025 May 28;20(5):e0323802. doi: 10.1371/journal.pone.0323802 (PMC12118823; doi:10.1371/journal.pone.0323802)

S1 Fig. Variation of the *kdr* genotypes across time


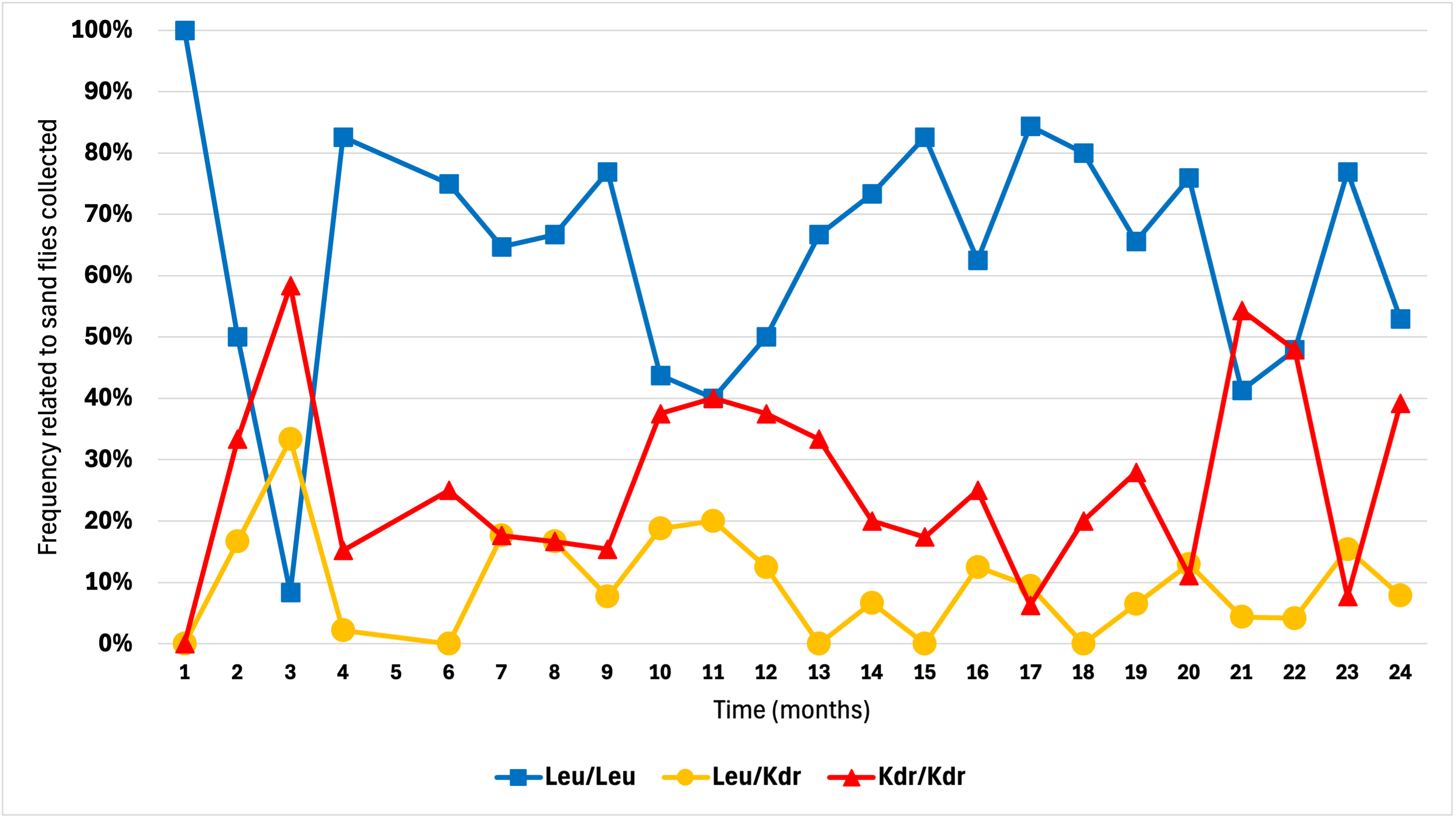


Note that no sand flies were collected in month 5.

Supplement: S1 Fig — Note that no sand flies were collected in month 5. (DOCX) [file pone.0323802.s004.docx]
